# Mesenchymal stem cells as a potential therapy for COVID-19

**DOI:** 10.1186/s13287-020-01678-8

**Published:** 2020-05-04

**Authors:** Shan Liu, Danyi Peng, Huijun Qiu, Ke Yang, Zhou Fu, Lin Zou

**Affiliations:** 1 Center for Clinical Molecular Laboratory Medicine/Newborn Screening Center National Clinical Research Center for Child Health and Disorders, Children’s Hospital of Chongqing Medical University, Chongqing, China; 2 Department of Respiratory Medicine; Ministry of Education Key Laboratory of Child Development and Disorders; China International Science and Technology Cooperation Base of Child Development and Critical Disorders, Children’s Hospital of Chongqing Medical University, Chongqing, China; 3 Chongqing Engineering Research Center of Stem Cell Therapy, Chongqing, China

**Keywords:** COVID-19, ALI, ARDS, MSCs

## Abstract

The outbreak of 2019 novel coronavirus disease (COVID-19) worldwide is becoming rapidly a major concern. The number of severe cases has increased dramatically worldwide, while specific treatment options are scarce. The main pathologic features of severe or critical COVID-19 were consistent with acute lung injure (ALI)/acute respiratory distress syndrome (ARDS), characterized by cellular fibromyxoid exudates, extensive pulmonary inflammation, pulmonary edema, and hyaline membrane formation. Mesenchymal stem cells (MSCs) can balance the inflammatory response and has been mentioned to be effective on ALI/ARDS from both infectious and noninfectious causes previously, presenting an important opportunity to be applied to COVID-19. In this commentary, we summarize the clinical trials of MSCs treatments on ALI/ARDS and raise MSCs as a hopefully alternative therapy for severe or critical COVID-19.

## Background

Severe acute respiratory syndrome coronavirus 2 (SARS-CoV-2) disease (COVID-19) is a newly-recognized infectious disease. It has rapidly transmitted and become a major concern all over the world. As of April 3, 2020, the total number of patients has risen sharply to 1,033,060 worldwide, with 54,677 (5.29%) deaths [1]. Apart from supportive care, oxygen supply in mild cases, and extracorporeal membrane oxygenation and low-dose corticosteroids in critical cases, intravenous remdesivir and convalescent plasma might be the effective potential therapy for SARS-CoV-2 infection. However, randomized clinical trials are needed to further evaluate the safety and efficacy of them in COVID-19 treatment. The specific and novel therapeutic methods for this disease are still being explored.

## Main text

The main pathologic features of severe or critical COVID-19 contain hypoxemia, diffuse alveolar damage with cellular fibromyxoid exudates, extensive pulmonary inflammation, pulmonary edema, and hyaline membrane formation. The pathologic changes are similar with acute lung injure (ALI)/acute respiratory distress syndrome (ARDS) [2], also observed in SARS and Middle Eastern respiratory syndrome (MERS) coronavirus infection. However, more serious inflammatory exudation, pulmonary edema and inflammatory cytokine storm, and milder pulmonary fibrosis and consolidation were observed in severe or critical COVID-19 than those in SARS. Mesenchymal stem cells (MSCs), which originate from bone marrow, fat, umbilical cord, placenta, and other tissues, have abundant supply, differentiation potential, powerful immunoregulation, and endogenous repair mechanisms. As one of the most widely studied adult stem cells in regenerative medicine, MSCs produce meaningful therapeutic outcomes for the treatment of pulmonary, cardiovascular, neurological, liver, and kidney diseases. The immune-regulation of MSCs depends mainly on modulating activation and effector function of immune cells, suppressing lung-infiltrated cells, and enhancing the resolution of pulmonary edema [3]. The incomplete revealed mechanisms but critical roles of MSCs on anti-inflammatory effects imply that MSCs is a potential therapy for severe and critical COVID-19.

MSCs have been identified to efficiently cure ALI/ARDS from both infectious and noninfectious causes, mediated primarily by paracrine mechanisms based on the released extracellular vesicles (EVs), such as microvesicles and exosomes [4]. In the cargo of the EVs, more than 850 unique gene products and more than 150 miRNAs have been identified by mass spectrometry analysis. Either the miRNAs or the transcripts are enriched in the regulators of the immune system [5, 6].

Detailed, MSCs can alter the behavior of both adaptive and innate immune cells. They can release keratinocyte growth factor, prostaglandin E2, granulocyte-macrophage colony-stimulating factor, and IL-6 and IL-13 to facilitate the phagocytosis and alternative activation of alveolar macrophage, alter the cytokine secretion profile of dendritic cell subsets, and decrease the release of interferon γ from natural killer cells. IL-10, transforming growth factor β, and tryptophan catabolizing enzyme indoleamine 2,3-dioxygenase secreted from them can also suppress the proliferation of T cells and change the cytokine secretion profile of T cell subsets [7]. Moreover, the proliferation, differentiation, and chemotactic properties of B cells were impaired by MSCs as well [8]. Except for the immune regulatory effects, MSCs can enhance restoration of capillary barrier [9], inhibit bacterial growth [10], and restore alveolar ATP [11]. All the functions mentioned above might also be effective on ARDS induced by COVID-19 infection.

Some clinical trials for evaluating the efficacy and safety of MSC treatment on ALI/ARDS have begun. The inclusion criteria are according to the Berlin definition of ARDS [12] in common, while the START trial [13] had a more strict definition of moderate-to-severe ARDS with 4 categories: (1) positive pressure ventilation by an endotracheal or tracheal tube with a PaO_2_/FiO_2_ ratio of < 200 with at least 8 cm H_2_O positive end-expiratory airway pressure, (2) bilateral infiltrates consistent with pulmonary edema on the frontal chest radiograph, (3) without clinical evidence of left atrial hypertension or a pulmonary arterial occlusion pressure ≤ 18 mmHg, and (4) categories 1–3 must be present within a 24-h time period and at the time of enrolment. Exclusion criteria included patients younger than 18 years, pre-existing severe disease of any major organs, pregnancy, malignant disease, severe chronic respiratory disease, recent deep vein thrombosis or pulmonary embolism, human immunodeficiency virus infection, or if informed consent could not be obtained. In addition, the patients in whom more than 96 h since first meeting the Berlin definition for ARDS had also been excluded in the START trial to avoid enrolling patients with late ARDS. The completed clinical trials demonstrate that MSCs are well tolerated without adverse effects in ALI/ARDS (Table 1) [14, 15]. Additionally, acute graft-versus-host-disease (GVHD) is a life-threatening complication of allogeneic hematopoietic stem cell transplantation due to its inflammatory storm. A meta-analysis revealed that infused MSCs could reduce acute GVHD grade and increase overall survival [16]. The therapeutic effects of MSCs on ALI/ARDS and GVHD with powerful inflammatory balance are solid proofs for the application of MSCs on other originated ALI/ARDS.

**Table 1:** The clinical trials of MSCs on ALI/ARDS

No.	Study name (NCT number)	Starting date	Phase	MSCs type and dose	The origin of ALI/ARDS	Key findings/study status
1	Adipose-derived MSCs in ARDS (NCT 01902082)	Nov 2012	Phase 1b	Intravenous infusion of human adipose MSCs, with 1 × 10^6^/kg	Unknown	Low dose of 1 × 10^6^ adipose-derived MSCs/kg was well tolerated
2	MSCs for ARDS (NCT 01775774)	Jul 2013	Phase 1b dose-escalation	Intravenous infusion of hBM MSCs, with 1, 5, or 10 × 10^6^/kg	Pneumonia or sepsis or aspiration or preeclampsia	All MSCs doses well tolerated No adverse effects detected
3	MSCs for treatment of ARDS in stem cell transplant patients (NCT 02804945)	Feb 2017	Phase 2	By vein with a maximum dose of 3 × 10^6^ cell/kg one time at day 1	Unknown	Completed
4	Clinical study to assess the safety and preliminary efficacy of HCR040 in ARDS (NCT 04289194)	Dec 2019	Phase 1–2	Intravenous administration of allogeneic adipose-derived adult MSCs expanded and pulsed with H_2_O_2_, the maximum tolerated dose (1 or 2 × 10^6^ cells/kg)	Unknown	Active, not recruiting
5	Repair of ARDS by stromal cell administration (NCT 03042143)	Jan 2019	Phase 1–2	Human UC-derived CD362-enriched MSCs, the maximum tolerated dose from phase 1 trial	Unknown	Recruiting
6	UC-MSCs therapy in ARDS (NCT 03608592)	Jun 2018	Not applicable	Intravenous infusion of UC-MSCs, dose 1 × 10^6^/kg	Unknown	Recruiting
7	UC-MSCs therapy in ALI (NCT 02444455)	May 2015	Phase 1–2	Intravenous infusion of hUB-MSCs, 5 × 10^5^ cell/kg once a day, three times	Unknown	Recruiting
8	MSCs in patients with ARDS (NCT 02112500)	Feb 2014	Phase 2	intravenously infusion of MSCs	Unknown	Recruiting
9	UC-MSCs in the treatment of novel coronavirus severe pneumonia (NCT 04273646)	Feb 2020	Not applicable	Intravenous 4 times of UC-MSCs (0.5 × 10^6^ UC-MSCs/kg BW) at day 1, 3, 5, 7	2019-COVID	Not yet recruiting
10	A pilot clinical study on inhalation of MSCs exosomes treating severe novel coronavirus pneumonia (NCT 04276987)	Feb 2020	Phase 1	5 times aerosol inhalation of MSCs-derived exosomes (2.0 × 10^8^ nano vesicles/3 ml) at day 1, 2, 3, 4, 5	2019-COVID	Not yet recruiting
11	UC-MSCs treatment for the 2019-novel coronavirus pneumonia (NCT 04269525)	Feb 2020	Phase 2	Intravenous infusion of UC-MSCs at day 1, 3, 5, 7	2019-COVID	Recruiting
12	Treatment with MSCs for severe corona virus disease 2019 (NCT 04288102)	Feb 2020	Phase 1–2	Intravenous 3 times of MSCs (BW ≥ 70 kg, 4.0 × 10^7^ cells/time; BW < 70 kg, 3.0 × 10^7^ cells/time) at day 0, 3, 6	2019-COVID	Not yet recruiting
13	MSCs treatment for pneumonia patients infected with 2019 novel coronavirus (NCT 04252118)	Jan 2020	Phase 1	Intravenous 3 times of MSCs 3.0 × 10^7^ at day 0, 3, 6	2019-COVID	Recruiting
14	Efficacy and safety of UC-MSCs for the treatment of severe viral pneumonia (NCT 04282928)	Feb 2020	Phase 1	Intravenous infusion of definitive HUC-MSCs (1 × 10^6^ cells/kg × BW (kg)	Influenza infection viral pneumonia	Not yet recruiting

Abbreviations: *MSCs* mesenchymal stem cells, *UC* umbilical cord, *UC-MSCs* umbilical-cord-derived mesenchymal stem cells, *hBM MSCs* human bone marrow-derived mesenchymal stem cells, *BW* body weight, *NCT* National Clinical Trial, *ALI* acute lung injure, *ARDS* acute severe respiratory failure, *COVID* nCoV infection severe pneumonia

Furthermore, MSC treatment significantly ameliorates ALI/ARDS induced by H9N2 avian influenza virus [17] and H5N1 [18] in mice, and even influenza virus in pig [19], indicating the possible efficacy of MSCs on viral ALI/ARDS. Importantly, MSCs can cure the patients with severe refractory ARDS [20], who failed to improve after both standard life support measures including mechanical ventilation and additional measures including extracorporeal ventilation, pointing that MSC could be used for serious viral ALI/ARDS. Some Chinese research groups triggered the clinical studies on MSCs treating critical COVID-19 (Table 1). In the trigged clinical trials, the inclusion criteria for severe or critical COVID-19 include respiratory rate (RR) ≥ 30 times/min, pulse oxygen saturation (SpO2) at rest ≤ 93%, partial pressure of PaO2/FiO2 ≤ 300 mmHg, requirement for mechanical ventilation, shock, etc. As of February 21, 2020, four patients with severe COVID-19 were recovered and discharged by MSCs therapy in China [21]. Therefore, we believe that MSCs would be a new effective therapeutic method for severe or critical COVID-19.

According to World Health Organization [22], the management of COVID-19 has mainly focused on infection prevention, case detection and monitoring, and supportive care. However, no specific anti-SARS-CoV-2 treatment is recommended because of the absence of evidence. Most importantly, the current guidelines emphasize that systematic corticosteroids should not be given routinely for COVID-19 treatment, which was also the recommendation in a Comment in The Lancet [23]. Evidence shows that MSCs can be used as a treatment without the occurrence of severe adverse events. In conclusion, it might be noteworthy to test the safety and efficacy of MSC transfusion in COVID-19 patients, especially for the severe or critical cases.

## Data Availability

Please contact us for the detailed data.

## Abbreviations

COVID-19: 2019 novel coronavirus disease
SARS-CoV-2: Severe acute respiratory syndrome coronavirus
ALI: Acute lung injure
ARDS: Acute respiratory distress syndrome
MSCs: Mesenchymal stem cells
MERS: Middle Eastern respiratory syndrome
GVHD: Graft-versus-host-disease
EVs: Extracellular vesicles
